# Occupational risk factors for sleep quality among Serbian airline pilots

**DOI:** 10.3389/fpubh.2024.1531523

**Published:** 2025-01-29

**Authors:** Marko Stojanović, Dejan Nesic, Miloš Maksimović, Zorica Terzić-Šupić, Jovana Todorović, Ivana Topalović, Željko Vlaisavljević, Jelena Ilić Živojinović

**Affiliations:** ^1^Faculty of Medicine, University of Belgrade, Belgrade, Serbia; ^2^Faculty of Medicine, Institute of Medical Physiology, University of Belgrade, Belgrade, Serbia; ^3^Faculty of Medicine, Institute of Hygiene and Medical Ecology, University of Belgrade, Belgrade, Serbia; ^4^Faculty of Medicine, Institute of Social Medicine, University of Belgrade, Belgrade, Serbia; ^5^College of Sports and Health, Belgrade, Serbia; ^6^Department of Gastroenterology and Hepatology, Clinical Center of Serbia, University of Belgrade, Belgrade, Serbia

**Keywords:** sleep quality, airline pilots, risk factors, occupational health, flight safety, stress management

## Abstract

**Introduction:**

The ability of airline pilots to maintain a good level of sleep goes a long way in ensuring operational effectiveness with regard to safety as well as personal health. The study assesses the risk factors for sleep quality of airline pilots in Serbia with the objective of determining those factors, both occupational and lifestyle that are paramount in assisting with sleep health.

**Methods:**

A cross-sectional study was conducted on a total of 66 Serbian commercial pilots, and the Pittsburgh Sleep Quality Index (PSQI) was used to assess their sleep quality. Demographic, occupational, lifestyle, biological and psychological variables were obtained through validated questionnaires. Multivariate logistic regression was used to determine the predictors of poor sleep quality (PSQI ≥5).

**Results:**

Overall sleep quality was poor in 65.2% of the participants. Significant factors associated with poor sleep quality included years of experience (OR = 1.17, *p* = 0.007) and levels of stress (OR = 2.87, *p* = 0.004). Particular lifestyle variables, including factors such as coffee intake, had initial relationships with sleep quality but were not significant in the multivariate models. With regard to age, a significant univariate association was also revealed but was dropped in the final model because of collinearity with years of experience.

**Conclusion:**

Serbian commercial pilots have a relatively high risk of inadequate sleep associated with years of experience and levels of stress. There is a need to implement organization-wide changes such as stress management schemes in order to improve sleep quality among pilots, and foster prioritizing well-being.

## 1 Introduction

The professional activity of a pilot is itself very demanding, as it includes responsibility for flight safety (1). Although the frequency of incidents is very low, the possible catastrophic consequences make operating a large passenger aircraft an inherently difficult task (2). In addition to experts from various fields, the responsibility for the safety of aircraft, passengers and crew rests mostly with pilots (2). This means that pilots must be in good health, which means annual medical examinations for license renewal in accordance with the requirements of the International Civil Aviation Organization (1). Irregular working hours, long periods on duty, as well as crossing time zones and insufficient opportunities for sleep are common working conditions of pilots, and can lead to disruption of the normal sleep/wake pattern and accompanying bodily functions (3). The short-term effects of such working conditions lead to digestive problems, fatigue, loss of sleep and reduced ability to work (4). Given that flight safety is critically affected by pilot sleep disorders (5). Sleep quality and recognition of risk factors for sleep disorders are of vital importance to all in the field of commercial civil aviation (6). The six main sleep disorders are insomnia, circadian rhythm disorders, sleep- disordered breathing, hypersomnia/narcolepsy, parasomnias, and restless legs syndrome, which can lead to a wide variety of dysfunction in most body systems, including endocrine, metabolic, higher cortical function, and neurological disorders (7). Previous research frequently cites the following risk factors for the development of sleep disorders among civilian pilots: age (8), years of experience (9), early flights (2, 10), shift work (8, 9, 11), and night shifts (8, 12).

There are several risk factors responsible for contributing to sleep disorders among pilots, depending on the type of flights they are involved in Reis et al. (10). In the case of long-haul pilots, the primary risk factors are considered to be performing night shifts and crossing multiple time zones (10) as circadian rhythms can influence their sleep–wake levels (13). However, in the case of short to medium-haul pilots, the main elements are flight spacing and extended hours on duty (10). For pilots of short-haul flights on aircraft models such as the Boeing 737 and 747, it has been found that in the 12 h before a flight, every hour of takeoff earlier than 9:00 AM results in a loss of 15 min of sleep (2). Additionally, an analysis using the PSQI (Pittsburgh Sleep Quality Index) questionnaire revealed a positive correlation between age and night flights with sleep disturbances in the largest study conducted in Asia among Chinese pilots (8). Older pilots often require more time to fall asleep, have less overall sleep, shorter REM (rapid eye movement) phases, and more awakenings during the night (9). In the field of aviation, where working in different shifts is something common, it has been noted that there is a high rate of sleepiness (13). In addition, working in shifts, especially ones that also involve the night period, causes disturbances to the biological clock and deteriorates the quality of sleep (12). Furthermore, a study that utilized actigraphy showed a significant negative correlation between years of experience and time spent in bed, as well as between the number of takeoffs and landings and levels of sleepiness (14). Other risk factors include screen use before bedtime, insufficient rest after flights, and the specific demands of working as a pilot in civil aviation (5).The studies highlight the importance of sleep disorders in pilots and their possible effects on flight safety. It has been shown in many other studies that this issue is still under-researched and so it is important to understand the risk factors for development of sleep disorders among civil pilots in the Republic of Serbia. This research, to the best of our knowledge, is the first such study in the country and seeks to shed lights on the risk factors and issues that pilots in our region encounter and also enhance the knowledge and management of sleep disorders in this profession.

## 2 Materials and methods

### 2.1 Study design and participants

The research was conducted at the Institute of Medical Physiology “Rihard Burijan,” Faculty of Medicine, University of Belgrade, in the period from 1st of April 2023 to 1st of April, 2024 as a cross-sectional study on a population of 66 pilots from the Republic of Serbia. All pilots signed informed consent and had the right to withdraw from the study at any time. The research was approved by the Ethics Committee of the Faculty of Medicine of the University of Belgrade under number 17/IX-9 dated September 5, 2023.

### 2.2 Inclusion and exclusion criteria

The study included male pilots over the age of 25, with a valid commercial flight license and employed full-time.

### 2.3 Sample size

A minimum sample size required to detect an effect size of 0.37 in a repeated measures analysis of variance with between-group factor for 2 groups and 2 repeated measures for the variables blood pressure, BMI, fat percentage, waist circumference, hip circumference, glucose, cholesterol, HDL, LDL level values and triglycerides for a statistical significance level of 0.05 and a statistical power of 0.90 is 60 patients. The size of the effect was obtained based on the assumed ratio of the value of explained and residual variance of 0.12 to 0.9. Due to the expected loss of respondents during the duration of the study of 20% for the final minimum sample size, the number 72 was taken. The calculation of the sample size was done using the program G-power 3.1.6 (http://www.psycho.uni-duesseldorf.de/abteilungen/aap/gpower3/download-andregister/Dokumente/GPower_3.1.6.zip).

### 2.4 Questionnaires

All pilots filled out questionnaires related to general information (age, position, marital status, smoking, etc.), eating habits, sleep quality, level of physical activity, as well as a questionnaire related to stress, anxiety and depression.

#### 2.4.1 General questionnaire

A general questionnaire included socio-demographic characteristics (age, professional education, marital status), years of experience, number of flying hours per week, job position - captain / first officer, family history of cardiovascular diseases.

#### 2.4.2 Specially designed questionnaire

A specially designed questionnaire addressed dietary habits, smoking, and consumption of alcohol, coffee, and energy drinks.

#### 2.4.3 Physical activity assessment

To determine the level of physical activity, we used the IPAQ (International Physical Activity Questionnaire), which was developed to assess physical activity across various countries for adults aged 18 to 65 years (15, 16). The long form of the IPAQ contains 27 questions covering different domains and collects data on job-related, transport-related, domestic, and leisure-time physical activities, as well as intensities (moderate, vigorous, walking) and includes sitting time (15, 16). The results of the IPAQ questionnaire are expressed in categories of low, moderate, and high levels of physical activity, or in MET-minutes per week (MET-minute represents the amount of energy expended through physical activity) (17).

#### 2.4.4 Assessment of depression, anxiety, and stress

To assess depression, anxiety, and stress, we used the Depression, Anxiety, and Stress Scale (DASS-42) (18), which has been validated in Serbia (19). This self-report instrument measures various symptoms of depression, anxiety, and stress over the past week with sufficient reliability (18, 20). The instrument consists of three subscales: depression, anxiety, and stress, each with 14 items, with responses ranging from 0- does not apply to me at all to 3- applies to me very much of most of the time (20).

#### 2.4.5 Assessment of sleep quality and disorders

To assess sleep quality and disorders, we used The Pittsburgh Sleep Quality Index (PSQI) (21), which has been validated in the Republic of Serbia (22). As a self-report test, the PSQI defines seven component scores related to: subjective sleep quality, sleep latency, sleep duration, habitual sleep efficiency, sleep disturbances, use of sleeping medication, and daytime dysfunction (21). The total score is calculated based on the sum of scores for these seven components and ranges from 0 to 21 (21, 23). With a sensitivity of 89.6% and specificity of 86.5%, a score of ≥5 indicates a distinction between good and poor sleep quality (21, 23).

### 2.5 Blood analysis and anthropometric measurements

Blood samples from all participants were analyzed by the same accredited laboratory. Anthropometric measurements were conducted to evaluate participants’ physical health and body composition.

#### 2.5.1 Blood analysis

Blood samples from all participants were analyzed to obtain values for glucose levels, total cholesterol, HDL, LDL, and triglycerides using the “SPFT” method with measurements expressed in mmol/L.

#### 2.5.2 Height

Height was measured using a medical stadiometer (Seca 206, Seca GmbH & Co. KG, Hammer Steindamm 9–25, 22,089 Hamburg, Germany, Made in China, Designed in Germany) with an accuracy of 0.1 cm.

#### 2.5.3 Body weight and body composition

Body weight, body fat percentage, muscle mass, and visceral fat index were measured using a medical digital scale (Tanita Inner Scan Body Composition Monitor BC-587, Tanita Corporation, Tokyo, Japan) with an accuracy of 0.1 kg and 0.1%. The body mass index (BMI) was calculated from the measured values of body weight and height.

#### 2.5.4 Waist and hip circumference

Waist and hip circumference were measured using a medical tape measure (Seca 201, Seca GmbH & Co. KG, Hammer Steindamm 3–25, 22,089 Hamburg, Germany, Made in China, Designed in Germany) with an accuracy of 0.1 cm. The waist-to-hip ratio was calculated from the measured waist and hip circumferences.

### 2.6 Statistical analysis

Depending on the type of variables and the normality of distribution, data description is presented as: frequency and percentage (n %), mean ± standard deviation, or median (min-max). The following statistical methods were used: t-test, Mann–Whitney test, Chi-square test, Fisher’s exact test. To model the relationship between the dependent variable (PSQI 5+ score) and independent variables, logistic regression was applied. Factors included in the multivariate regression model were those that were statistically significant in univariate analyses at a significance level of 0.05. Statistical hypotheses were tested at a significance level (alpha) of 0.05. Results are presented in tables and figures. All data were processed using IBM SPSS Statistics 24 (SPSS Inc., Chicago, IL, USA) software or the R programming environment (R Core Team, 2023).

## 3 Results

The study included 66 participants, and their average age was 41.6 ± 8.0 years. The mean PSQI score of the participants was 5.7 ± 2.5, with the median PSQI of 5. The participants were divided into two groups according to their Pittsburgh Sleep Quality Index (PSQI), out of which, 43 participants (65.2%) had a PSQI score of ≥5 which indicated poor sleep quality, while 23 participants (34.8%) had a PSQI score of <5 indicating good sleep quality. Table 1 includes age, marital status, and educational level; years of experience; job position and hours spent on flights in a week. There were significant differences between the group with PSQI 5+ or PSQI <5 in the mean age, years of experience and job position.

**Table 1:** Demographic and occupational characteristics of participant.

Variables	Total (*n* = 66)	PSQI 5 + (*n* = 43)	PSQI < 5 (*n* = 23)	*p*-value
Age, mean ± sd	41.6 ± 8.0	43.2 ± 8.1	38.6 ± 6.8	0.024*
Marital Status, *n* (%)				0.829
Married	47 (71.2%)	31 (72.1%)	16 (69.6%)	
Other	19 (28.8%)	12 (27.9%)	7 (30.4%)	
Level of Education, *n* (%)				0.144
College	22 (33.3%)	17 (39.5%)	5 (21.7%)	
University	44 (66.7%)	26 (60.5%)	18 (78.3%)	
Years of Experience, median (range)	15 (1-31)	19 (1-31)	10 (1-22)	0.001*
Job Position, *n* (%)				0.012*
Captain	47 (71.2%)	35 (81.4%)	12 (52.2%)	
First Officer	19 (28.8%)	8 (18.6%)	11 (47.8%)	
Weekly Flight Hours, mean ± sd	18.22 ± 3.58	18.5 ± 3.6	18.1 ± 3.6	0.688

The characteristics of both groups are presented in Table 1.

The factors in the Table 2 include the use of cigarettes, coffee, energy drinks, beer, wine, and hard liquor use. There was a significant difference between the groups in the frequency of coffee consumption (*p* = 0.008). Other variables such as current smoking status (*p* = 0.692), consumption of energy drinks (*p* = 0.284), beer intake (*p* = 0.706), wine intake (*p* = 0.663), and liquor intake (*p* = 0.149) were not statistically significant between the PSQI groups.

**Table 2:** Lifestyle factors.

Variables	Total (*n* = 66)	PSQI 5 + (*n* = 43)	PSQI < 5 (*n* = 23)	*p*-value
Cigarette Use, *n* (%)				0.692
Never	38 (57.6%)	24 (55.8%)	14 (60.9%)	
Yes	28 (42.4%)	19 (44.2%)	9 (39.1%)	
Coffee Consumption				0.008*
Every day	46 (69.7%)	35 (81.4%)	11 (47.8%)	
2-3 times a week	8 (12.1%)	3 (7.0%)	5 (21.7%)	
4-6 times a week	3 (4.5%)	1 (2.3%)	2 (8.7%)	
Once a week	1 (1.5%)	0 (0.0%)	1 (4.3%)	
Never/Once or a few times a year	8 (12.1%)	4 (9.3%)	4 (17.4%)	
Energy Drink Consumption, *n* (%)				0.284
Never, less than 1 per month	52 (78.8%)	32 (74.4%)	20 (87.0%)	
1-3 per week	13 (19.7%)	11 (25.6%)	2 (8.7%)	
1 per day	1 (1.5%)	0 (0.0%)	1 (4.3%)	
Beer Consumption, *n* (%)				0.706
Never	12 (18.2%)	8 (18.6%)	4 (17.4%)	
Once a week or less, small quantities (one glass)	25 (37.9%)	17 (39.5%)	8 (34.8%)	
Once a week or less, large quantities (more than three glasses)	13 (19.7%)	9 (20.9%)	4 (17.4%)	
More than once per week, small quantities (one glass)	12 (18.2%)	5 (11.6%)	7 (30.4%)	
More than once per week, large quantities (more than three glasses)	2 (3.0%)	2 (4.7%)	0 (0.0%)	
Daily, small quantities (one glass)	2 (3.0%)	2 (4.7%)	0 (0.0%)	
Wine Consumption, *n* (%)				0.663
Never	13 (19.7%)	9 (20.9%)	4 (17.4%)	
Once a week or less, small quantities (one glass)	33 (50.0%)	19 (44.2%)	14 (60.9%)	
Once a week or less, large quantities (more than three glasses)	8 (12.1%)	7 (16.3%)	1 (4.3%)	
More than once per week, small quantities (one glass)	12 (18.2%)	8 (18.6%)	4 (17.4%)	
Hard Liquor Consumption, *n* (%)				0.149
Never	23 (34.8%)	12 (27.9%)	11 (47.8%)	
Once a week or less, small quantities (one glass)	24 (36.4%)	17 (39.5%)	7 (30.4%)	
Once a week or less, large quantities (more than three glasses)	8 (12.1%)	6 (14.0%)	2 (8.7%)	
More than once per week, small quantities (one glass)	8 (12.1%)	6 (14.0%)	2 (8.7%)	
More than once per week, large quantities (more than three glasses)	2 (3.0%)	2 (4.7%)	0 (0.0%)	
Daily, small quantities (one glass)	1 (1.5%)	0 (0.0%)	1 (4.3%)	

Table 3 summarizes the biochemical and anthropometric characteristics of participants (*n* = 66), categorized by PSQI scores (≥5, *n* = 43; <5, *n* = 23). There were no differences between the groups in cholesterol (5.0 ± 1.0 vs. 5.1 ± 1.1 mmol/L; *p* = 0.135), triglycerides (median 1.1 mmol/L; *p* = 0.731), HDL (1.4 ± 0.3 mmol/L; *p* = 0.988), or LDL (3.0 ± 0.9209 mmol/L; *p* = 0.130). There was also no significant difference in mean glucose (5.4 ± 0.9 mmol/L; *p* = 0.191) and BMI (25.8 ± 2.7; *p* = 0.342). Median visceral fat was 8 (range 1–16; *p* = 0.060). Regarding medication use, 87.9% reported no antihypertensive medications (*p* = 0.244) and 86.4% reported no other medications (*p* = 0.146), with no significant differences. There was a statistically significant difference in stress levels (*p* = 0.016). Higher percentages of normal stress levels among participants with PSQI<5 where observed (60.9%) in contrast to the PSQI≥5 group (27.9%). There were significant differences in the frequencies of the categories of DASS stress scale between the groups. The differences between the groups on domains of DASS scale and IPAQ are presented in Table 4. All the variables which were shown significant were entered in the multivariate logistic regression model with PSQI5+ score as an outcome variable.

**Table 3:** Biochemical and anthropometric characteristics.

Variables	Total (*n* = 66)	PSQI 5 + (*n* = 43)	PSQI < 5 (*n* = 23)	*p*-value
Cholesterol, mean ± sd	5.0 ± 1.0	5.1 ± 1.1	4.7 ± 0.8	0.135
Triglycerides, median (range)	1.1 (0.4–4.4)	1.1 (0.4–4.4)	1.1 (0.5–2.8)	0.731
HDL, mean ± sd	1.4 ± 0.3	1.4 ± 0.3	1.4 ± 0.2	0.988
LDL, mean ± sd	3.0 ± 0.9	3.2 ± 1.0	2.8 ± 0.8	0.130
Glucose, mean ± sd	5.4 ± 0.9	5.5 ± 1.1	5.2 ± 0.3	0.191
BMI, mean ± sd	25.8 ± 2.7	26.0 ± 2.9	25.3 ± 2.3	0.342
Waist-to-hip ratio, mean ± sd	0.9 ± 0.1	0.9 ± 0.1	0.9 ± 0.1	0.209
Body fat, mean ± sd	21.2 ± 3.6	21.4 ± 3.6	20.8 ± 3.7	0.597
Muscle mass, mean ± sd	63.7 ± 6.3	64.4 ± 6.9	62.5 ± 4.9	0.232
Water percentage, mean ± sd	53.6 ± 2.8	53.2 ± 2.8	54.3 ± 2.7	0.155
Visceral fat, median (range)	8 (1–16)	8 (1–16)	7 (1–13)	0.060
Antihypertensive medications, *n* (%)				0.244
No	58 (87.9%)	36 (83.7%)	22 (95.7%)	
Yes	8 (12.1%)	7 (16.3%)	1 (4.3%)	
Other medications				0.146
No	57 (86.4%)	35 (81.4%)	22 (95.7%)	
Yes	9 (13.6%)	8 (18.6%)	1 (4.3%)	

**Table 4:** Physical activity levels, depression, anxiety, and stress.

Variables	Total (*n* = 66)	PSQI 5 + (*n* = 43)	PSQI < 5 (*n* = 23)	*p*-value
IPAQ level PA. *n* (%)				0.636
Low	39 (59.1%)	24 (55.8%)	15 (65.2%)	
Moderate	14 (21.2%)	11 (25.6%)	3 (13.0%)	
High	13 (19.7%)	8 (18.6%)	5 (21.7%)	
DASS_ depression				0.068
Normal	48 (72.7%)	28 (65.1%)	20 (87.0%)	
Mild	8 (12.1%)	7 (16.3%)	1 (4.3%)	
Moderate	9 (13.6%)	7 (16.3%)	2 (8.7%)	
Severe	1 (1.5%)	1 (2.3%)	0 (0.0%)	
Extremely severe	0 (0.0%)	0 (0.0%)	0 (0.0%)	
DASS_ anxiety				0.771
Normal	53 (80.3%)	34 (79.1%)	19 (82.6%)	
Mild	4 (6.1%)	3 (7.0%)	1 (4.3%)	
Moderate	3 (4.5%)	2 (4.7%)	1 (4.3%)	
Severe	4 (6.1%)	3 (7.0%)	1 (4.3%)	
Extremely severe	2 (3.0%)	1 (2.3%)	1 (4.3%)	
DASS_stres				0.016*
Normal	26 (39.4%)	12 (27.9%)	14 (60.9%)	
Mild	17 (25.8%)	13 (30.2%)	4 (17.4%)	
Moderate	17 (25.8%)	13 (30.2%)	4 (17.4%)	
Severe	5 (7.6%)	4 (9.3%)	1 (4.3%)	
Extremely severe	1 (1.5%)	1 (2.3%)	0 (0.0%)	

**Figure 1:**
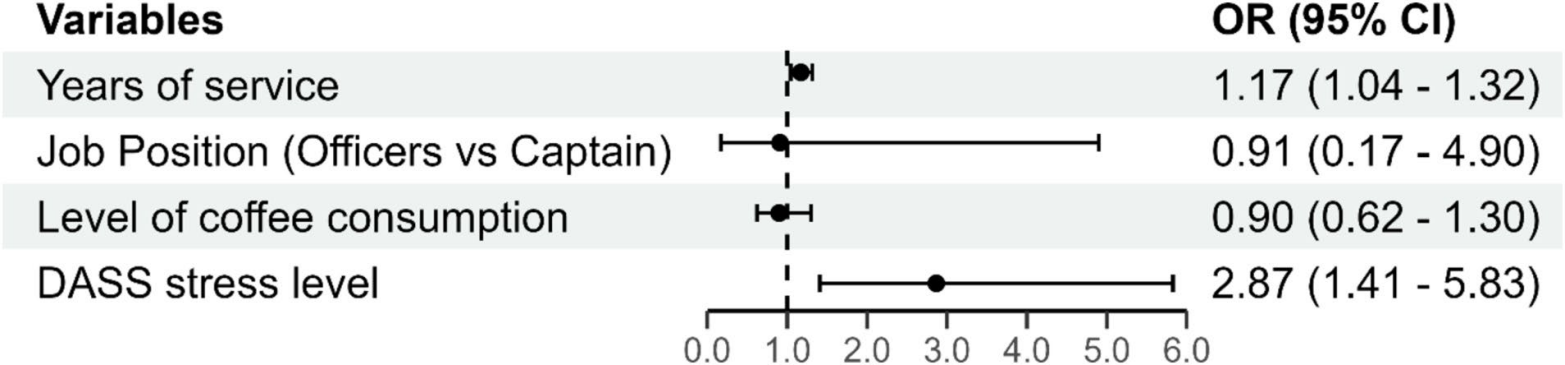
The multivariate logistic regression model.

Due to co-linearity with the variable years of experience, the variable age was not included in the multivariate model. The results of the multivariate logistic regression are presented in Figure 1.

The overall model (including all predictors) was statistically significant (Chi-square = 23.395; DF = 4; *p* < 0.001) and explains 41% of the variation in the outcome variable. There is no significant multicollinearity among the variables.

In the multivariate logistic regression model (Figure 1), the factors associated with PSQI5+ score were: Years of Service (*B* = 0.159; *p* = 0.007), with an odds ratio (OR) of 1.17. This indicates that for each additional year of experience, pilots have a 17% higher chance of having a PSQI 5+ score, controlling for all other factors in the model. DASS Stress Level (*B* = 1.053; *p* = 0.004), with an odds ratio (OR) of 2.87. This suggests that for each increase in the level of stress, pilots have nearly 3 times higher odds of having a PSQI 5+ score, controlling for all other factors in the model.

## 4 Discussion

The results of this study emphasize the prevalence of sleep disorders in the Serbian airline pilots’ population as concerning, given that 65.2% of the respondents obtained scores of 5 and above on the Pittsburgh Sleep Quality Index, which is an indication of poor quality of sleep, whereas only 34.8% scored below 5, indicating good quality of sleep. This echoes sentiments of irritable sleep patterns in pilots from previous studies. For example, almost 63% of aircrew officers reported sleep disorders in Monin et al. (5), demonstrating provocative levels of sleep deprivation among the subjects. In addition, Roach et al. (2) observed that, due to their early scheduled duty hours, more than half (62%) of the short-haul pilots managed to sleep less than enough. Notably, Abdelaziz et al. (14) tagged along with Saudi Arabian pilots in their study and found a low prevalence (33%) of poor sleep quality but 66.7% had irregular sleep cycles. The stressful nature of airline operations, characterized by variable work schedules and night flights, has been extensively studied, and it has been established that this type of operations is very disruptive to pilots’ normal sleeping cycle. They found a significant negative correlation between years of experience and time in bed, as well as negative correlation between the number of takeoffs and landings per month and sleepiness, which was difficult to explain. This study examines the various determinants of sleep quality among airline pilots, revealing age years of service, job position, coffee consumption, and stress as among the key factors. The negative relationship between age, years of experience, and sleep quality is most probably due to the physical and mental strain that years of working under erratic hours and heavy workloads without rest brought about. In the univariate analysis, longer years of service showed a strong link to poorer sleep quality, while in the multivariate model, it remained one of the most significant predictors. In other words, risk of higher PSQI increased by 17% with each year of practice experience while controlling for other factors. In a study among 1,208 Chinese airline pilots by Shi et al. (8), however, it was found that with increasing years of exposure to night flights, the risk of sleep disturbances owing to employment-related stress elevated. The higher risk of sleep disorders in pilots aged ≥45 years was significantly associated the mean monthly night flight duration of ≥30 h in previous 3 years. Their findings suggest that night flights may cause more sleep problems in senior pilots because they are associated with circadian rhythm disturbances. Similarly, Monin et al. (5), in a French population found that the negative impact of night flights on sleep was acute with increasing age and increased length of time at night respondents worked and this was primarily due to cumulative stressors. Hierarchical status of job position was a variable that appeared correlated with sleep quality (PSQI ≥5) in univariate analysis, with higher-rank officers suffering from more sleep problems than their juniors, as they had more responsibilities and pressure. This finding is concurrent with the work of Alaminos-Torres et al. (11) studied Spanish commercial airline pilots and identified that senior pilots were more overworked and tired, thus suffered more sleep interferences. This research showed that it was sleepiness, but also work overload and total hours of work, were the most important predictors of fatigue. Nevertheless, hierarchical status of job position was not a significant predictor in multivariate analysis, indicating that probably years of working, cumulative wear-out effects and other factors are more critical to sleep quality. In line with this, Kim and Choi (23) revealed that stress coping mechanisms and support systems differ from role to role and this is why solutions to role of stress include relief of job sleep disorder through a holistic approach only. Their results showed that quality of life and physical activity were key preventive factors, as higher quality of life was associated with an 84% reduction in fatigue odds, and vigorous physical activity with an 18% decrease in the odds. Quality of life includes not only physical and mental health, but also social relationships and the environment. There was no clear association between the weekly flight hours and the quality of sleep in both the univariate and multivariate analyses, which implies that the total number of hours flown per week may have no direct relationship with the sleep patterns of the pilots. This observation is different from those made by Roach et al. (2), who claimed that longer flight hours contributed to pilot fatigue and dysfunctional sleep, particularly in short-haul pilots where take-off and landing was more frequent. In the same vein, Sallinen et al. (6), showed that total hours which also included irregular hours could interfere with sleep by reducing recuperation time. However, the current study did not show any significant association because it appears that for this group of study participants, other aspects such as the timing of shifts, rest breaks, and personal stress levels are more pertinent to sleep quality than the total number of hours worked per week alone. It indicates that strategies targeted at improving sleep such as scheduling resumption of work to more than that of deregulation of flight hours may be more efficient. Univariate analyses established a strong correlation between coffee consumption and poor sleep quality. However, this was not the case with the multivariate models. Caffeine was also reported to be used by pilots to reduce tiredness but the sleep disruption it caused was related more to long hours of work and demanding schedules in Finland Sallinen et al. (6). Early starts and late finishes led to increased coffee drinking as a strategy to cope with inadequate sleep according to Australian researchers Roach et al. (2). Similary, Monin et al. (5) in their research carried out in France, made a connection between sleep problems and consumption of caffeine. However, it is probably more a question of stress levels than caffeine itself. It has also been shown that as much as caffeine may help in postponing drowsiness, this is never the case when stress from the workplace and heavy workload come into play, thus, there is a necessity for an approach to fatigue management, which looks into the whole lifestyle and work-related stressors as well Zaslona et al. (13). Biochemical markers analyzed using cholesterol and triglycerides or BMI in relation to sleep quality did not produce significant relationships, indicating that a person’s social and behavioral lifestyle may be more immediate causes for poor sleep experienced. This is in agreement with the findings of Alaminos-Torres et al. (11), who stated that in pilots the quality of sleep is more associated with behavioral and psychosocial issues such as work overload and fatigue rather than physiological parameters. In their study of Spanish commercial airline pilots, they also identified links between workload, fatigue, and daytime sleepiness, which highlights the need to consider these variables in any sleep research conducted on aviation personnel. A fundamental cause for poor sleep quality was exposed as stress levels, with more stress correlating to more sleep problems, highlighting the extreme sociopsychological situations that airline pilots are subjected to. Data suggest that with an increase in stress level, the likelihood of scoring a PSQI ≥5 among pilots increases by almost three times, after controlling for all other variables. Such an effect corresponds to the effect highlighted in a study by Van Drongelen et al. (3), which studied the effects of work-related stress on the level of alertness in pilots and found it to be a major cause of such fatigue and the inability to concentrate safely, which builds over time. Other researchers, such as Sallinen et al. (6) have also noted that it is the stress that results from working irregular duty schedules and long operative periods that increases fatigue, disrupts sleep and interferes with the pilots’ alertness during both short and long-haul flights. Therefore, it is necessary to propose measures that directly address stressors such as stress management and resilience training so as to optimize sleep health and general health among aviators. The research evaluated the depressive and anxious tendencies, within the pilot group, with the result that a majority of the individuals scored at the normal levels of depression, with only a few presenting mild to moderate symptoms. The lack of strong correlation between mental-health related symptoms and sleep disturbance is consistent with the findings of other studies, e.g., those conducted by Monin et al. (5) who, while evaluating a sample of 2.000 French aircrew, found that 20% of the responders suffered mild psychological distress despite the fact that these symptoms did not correlate with disturbance of their sleep in general. Likewise, Shi et al. (8) noted that while pilots had psychological factors, they were not the major determinants of the sleep, as older age and longer night flying time were more contributory. These outcomes indicate that for pilots, somatic risk factors such as work-related stress and tiredness may lower the quality of sleep more than the psychological symptoms. Application of International Physical Activity Questionnaire (IPAQ) showed no significant correlation between sleep quality and physical activity levels in the sampled pilots of this study. This is in agreement with the observations of Monin et al. (5) and Reis et al. (12). Although moderate physical activity tends to be associated with better sleep quality in the general population, the same cannot be said for pilots, as the benefits of such activities may be outweighed by more pressing matters, such as irregularity in schedules and exhaustion from work. Nevertheless, research by van Drongelen et al. (4) has established that when pilots engage in more exercise and support measures focused on combating fatigue and stress are also provided, sleep quality can be improved. Therefore, such approaches will be explored in future studies to examine possible interactions. In this way, while physical activity is less likely to be detrimental, in the case of sleep quality, the effect of the sole activity may be too deficient in aviation personnel, whose practice seems to be constrained more by social and work environment than as an individual.

## 5 Limitations and future research

Although this study included a large number of Serbian commercial pilots, this is the first such study in our country on this population. Further research needs to be directed toward controlling the implementation of preventive measures and interventions to reduce stress and sleep-related problems.

## 6 Conclusion

The research reveals that Serbian airline pilots suffer from poor sleep quality which is attributable to the number of years in service and high levels of stress among other factors. Every year in service added the chance of poor sleep by 17% while the control of stress factors increased the risk by almost three folds. Lifestyle risks including coffee drinking were shown to have some degree of correlation with sleep quality, however, work related stress was found to be the main factor. Consequently, it becomes apparent that there is a need to implement specific strategies within the aviation sector, in particular stress management interventions and alternative rosters that consider years of experience in order to promote pilot’s health and enhance flight safety. Positively addressing the risk factors will lead to a healthier and more resilient workforce in the aviation sector, thereby improving the sustainability of operations. Endeavors aimed at the health of pilots are important not only for safety but for preventing the indirect costs that will arise in future due to absenteeism, staff turnover, and interruptions of operations which in the end will help in achieving responsible, more safe and sustainable aviation.

## Data Availability

The raw data supporting the conclusions of this article will be made available by the authors, without undue reservation.
